# The combined effect of parathyroid hormone (1–34) and whole-body vibration exercise on physical performance in OSteoporotic women (PaVOS study): a secondary analysis from a randomised controlled trial

**DOI:** 10.1186/s13102-020-00204-w

**Published:** 2020-09-05

**Authors:** Ditte Beck Jepsen, Tahir Masud, Anders Holsgaard-Larsen, Stinus Hansen, Niklas Rye Jørgensen, Jesper Ryg

**Affiliations:** 1 Department of Geriatric Medicine, Odense University Hospital, Odense, Denmark; 2 Department of Clinical Research, University of Southern Denmark, Odense, Denmark; 3 Department of Geriatric Medicine, Nottingham University Hospitals Trust NHS, Nottingham, UK; 4 Department of Orthopaedics and Traumatology, Odense University Hospital, Odense, Denmark; 5 Department of Endocrinology, Hospital South West Jutland, Esbjerg, Denmark; 6 Department of Regional Health Research, University of Southern Denmark, Odense, Denmark; 7 Department of Clinical Biochemistry, Rigshospitalet, Copenhagen, Denmark; 8 OPEN- Odense Patient data Explorative Network, Odense University Hospital/ University of Southern Denmark, Odense, Denmark

**Keywords:** Exercise, Osteoporosis, Physical performance, Short physical performance battery, Whole-body vibration, Women

## Abstract

**Background:**

The aim of this study was to investigate the effect on physical performance of combining whole-body vibration exercise (WBV) with parathyroid hormone 1–34 (teriparatide) compared to teriparatide alone.

**Methods:**

A secondary analysis from a RCT where postmenopausal women with severe osteoporosis were randomised to WBV plus teriparatide (intervention) or teriparatide alone (control). WBV was applied three times/week (6x1min WBV:1 min rest, (peak acceleration 3.6 *g*)) for twelve months. Both groups received teriparatide 20 μg s.c./day. The primary endpoint (bone mineral density) is reported elsewhere. Physical performance measures (Short Physical Performance Battery (SPPB), Timed-Up-and-Go (TUG), leg extension power, and grip strength) were obtained at baseline, three-, six-, and twelve months, lean mass at baseline and twelve months. Data were analysed with mixed linear regression model or robust cluster regression in an intention to treat analysis.

**Results:**

Thirty-five women aged (mean ± SD) 69 ± 7) years were recruited of which thirty-two (91%) completed the twelve months follow-up (WBV + teriparatide = 15, teriparatide = 17). SPPB score (mean ± SD) improved significantly at three months in the WBV + teriparatide group from 9.13 ± 2.03 to 10.35 ± 1.69 (*p* = 0.014) with a statistical trend towards a between-group change in favor of the WBV + teriparatide group (0.86 [95%CI(− 0.05,1.77), *p* = 0.065]). Both groups improved in leg extension power during the study period whereas no changes were seen in TUG, grip strength, or lean mass in either group. No statistical significant between-group differences were observed.

**Conclusion:**

WBV may improve some short-term aspects of physical performance in severely osteoporotic postmenopausal women who are receiving teriparatide treatment.

**Trial Registration:**

ClinicalTrials.gov, ID:NCT02563353

## Background

Osteoporosis and increased propensity to fall are major determinants of fragility fractures in an aging population. The economic burden of such fractures is high [1] and expected to rise with increasing life expectancy [2]. Moreover, the individual burden of a fragility fracture is substantial with decreased quality of life, risk of dependence, and increased mortality [3, 4]. Several pharmaceutical agents are approved for the treatment of osteoporosis and have been shown to reduce the risk of fragility fractures [5, 6]. The anabolic agent parathyroid hormone analogue 1–34 (teriparatide) is capable of increasing bone mineral density (BMD) in lumbar spine and proximal femur in postmenopausal women with osteoporosis [7]. The risk of falling is multifactorial with decreased neuromuscular function and impaired balance being prominent risk factors [8]. Exercise programs that challenge balance can reduce fall rates in community-dwelling older adults and those with higher training intensity yielding greater effects [9]. Whole-body vibration exercise (WBV) has emerged as an alternative training intervention that is well tolerated in women with osteoporosis and even in older adults with a high risk of falls [10, 11]. WBV has the capacity to improve muscle strength of the lower extremities and body balance in older adults, both important factors in decreasing the risk of falls [12–14]. A previous study has shown a positive effect of combining osteoporotic treatment i.e. alendronate and exercise, where alendronate increased bone mass, exercise improved the participants’ muscular performance and dynamic balance, potentially decreasing the risk of fractures [15].

We have previously shown that combining pharmacological treatment for osteoporosis in the form of teriparatide with WBV as an exercise increased BMD of lumbar spine by an additional 2.95% [16]. If in addition WBV also can increase physical performance, then this could potentially result in an optimized fracture prevention intervention. However, the potential combined effect of this dual approach on physical performance is unknown.

Therefore, the aim of this pre-defined secondary analysis from a 12 month randomized controlled trial (RCT) was to investigate the effect on physical performance of combining WBV with teriparatide treatment compared to teriparatide alone in postmenopausal women with severe osteoporosis.

## Methods

This study is a sequel analysis of a RCT [17]. Outcome measures for the present analysis were a priori defined in a statistical analysis plan at ClinicalTrials.gov (NCT02563353) and in the published study protocol [17]. Data collection for this study started November 2015 and was finalized November 2018 and study results are reported according to the CONSORT statement [18].

### Participants

The recruitment of women starting teriparatide was performed in five outpatient clinics: the Osteoporosis Clinic or Department of Geriatric Medicine (Odense University Hospital), Department of Endocrinology (Hospital South West Jutland), Department of Endocrinology (Hospital Lillebaelt), or Department of Endocrinology and Internal Medicine (Aarhus University Hospital). Inclusion criteria were: postmenopausal women ≥50 years of age, with either one vertebral fracture within the last 3 years with > 25% reduction in vertebral height and BMD at the lumbar spine or total hip with T-score < − 3, or at least two vertebral fractures with > 25% reduction in vertebral height, with no requirements for low BMD, planned to start treatment with teriparatide. Exclusion criteria were: ongoing oral glucocorticoid treatment, inability to tolerate WBV for 1 min at screening, not being able to give informed consent, or contraindications to WBV i.e. pacemakers and knee or hip joint replacements.

### Design and randomization

A 12 month, superiority- RCT with two parallel arms investigating the effect of combined WBV and teriparatide compared to teriparatide alone was conducted from November 2015 to November 2018. The investigator who performed the web-randomization was not involved in outcome assessment. Sham WBV was not utilized, but the assessors performing the outcome measurements were blinded to group assignment. A more detailed description can be found elsewhere in the published study protocol [17].

All participants received subcutaneous teriparatide 20 μg/day. Web-based block randomization was conducted at baseline after eligibility criteria were satisfied and the baseline measurements were obtained.

### Intervention and adherence

The WBV was performed on a Power plate My5 (Power plate®, UK), using a protocol that was well tolerated in a previous study in frail older individuals with a frequency of 30 Hz and amplitude of 1 mm (low displacement) with peak acceleration of 35.53 m s^− 2^ rms (3.6 *g*) [10]. The training was conducted at the participants own homes and consisted of up to 12 min of training with the WBV:rest ratio 1:1 min, including 6 min of vibration, 3 days a week with 1 day pause in between. The training progressed the first 8 weeks up to the 6 min of vibration to ensure that participants could tolerate the training in the total 12 months. The training protocol followed the safety ISO-guidelines [19] and has previously been shown to be safe in a population of older adults aged 80 years in term of falls risk [10]. All participants trained without shoes. They were instructed at home and performed their first training supervised. The participants were followed with monthly telephone calls and were instructed to contact the project manager if they needed clarification or had questions. Where necessary the instructor re-visited the participants to ensure that the training was conducted as instructed. The protocol and monitoring by attendance log has previously been described elsewhere [17]. High adherence to WBV was defined as being > 75% adherent (i.e. more than 685 exercise sessions out of 913). Falls were assessed through calendars and monthly telephone contact was used to ensure adherence and to collect data on pain, dizziness, falls, and other adverse events. Physical activity was assessed using the international physical activity questionnaire (IPAQ) [20].

### Outcomes

The primary outcome of BMD is reported elsewhere [16]. This study reports predefined secondary outcomes of physical performance, muscle function, and lean mass measures.

Included measures were: Short Physical Performance Battery (SPPB) [21], Timed-Up-and-Go test (TUG), leg extensor power [22], and hand grip strength [23]. SPPB consists of a five times sit to stand test (5TSTS), a three meter walking test and a tandem test. SPPB was scored according to guidelines and calculated using the points score validated to assess lower extremity function and balance in older adults [21]. The time used to perform the TUG was recorded for the participants to rise from a chair, walk three meters, return and sit back down, with three repetitions. Unilateral leg extension power was assessed using a Nottingham leg extensor power rig, (Medical Engineering Unit, School of Biomedical Sciences, University of Nottingham Medical School, Nottingham, UK) measuring peak extension power [22]. Hand grip strength was measured using a Smedley dynamometer (TTM; Tokyo, Japan). The measurements were done according to standard protocol, using the peak values from the bilateral testing [23].

The physical tests were performed at baseline, three-, six-, and 12 months of follow-up by trained technicians using a standardized protocol. With repeated measurements the participants’ best test score was applied. Reported coefficients of variation for TUG, 5TSTS, and leg power were 5.3, 5.1, and 9%, respectively [22, 24].

Whole body dual-energy X-ray absorptiometry (DXA) (Hologic Discovery, Waltham, MA, USA) was performed to assess muscle mass at baseline and 12 months. Appendicular lean mass was determined using the equation: appendicular lean mass (kg)/height (m)^2^.

### Statistical methods

The sample size was calculated from expected changes in the primary outcome of BMD [17]. Allowing for 20% drop out rate the inclusion of 40 participants was planned. Power and sample size considerations for this follow-up study on secondary outcomes were evaluated a posteriori.

Data were presented with means and standard deviations or medians and interquartile ranges depending on distribution. Between-group differences in baseline characteristics and study adverse events were assessed using the Student t-test, Wilcoxon rank sum, or the chi-square test according to continues outcomes with normal or non-normal distributions, or categorical outcomes respectively. The treatment groups were compared using a linear mixed-effect model with fixed effects for treatment groups at baseline, time, and time by treatment-group interaction. Data that had non-normal distributed residuals were analyzed in a linear regression model with robust cluster estimation with clusters to deal with the individuals’ effect on the repeated measurements. Model assumptions were checked using histograms, plots of the residuals against fitted values, and normal probability plots of the residuals. The primary mixed multilevel model included all observed values, whereas a secondary analysis applied multiple imputations to missing values. A significance level of 0.05 was chosen. All statistical analyzes were performed using the Statistical Package STATA (version 14 and 15, Stata Corp., College Station, TX, USA). Intention to treat (ITT) and per protocol (PP) analysis were conducted to investigate if adherence (> 75%) affected the results.

## Results

**Fig. 1:**
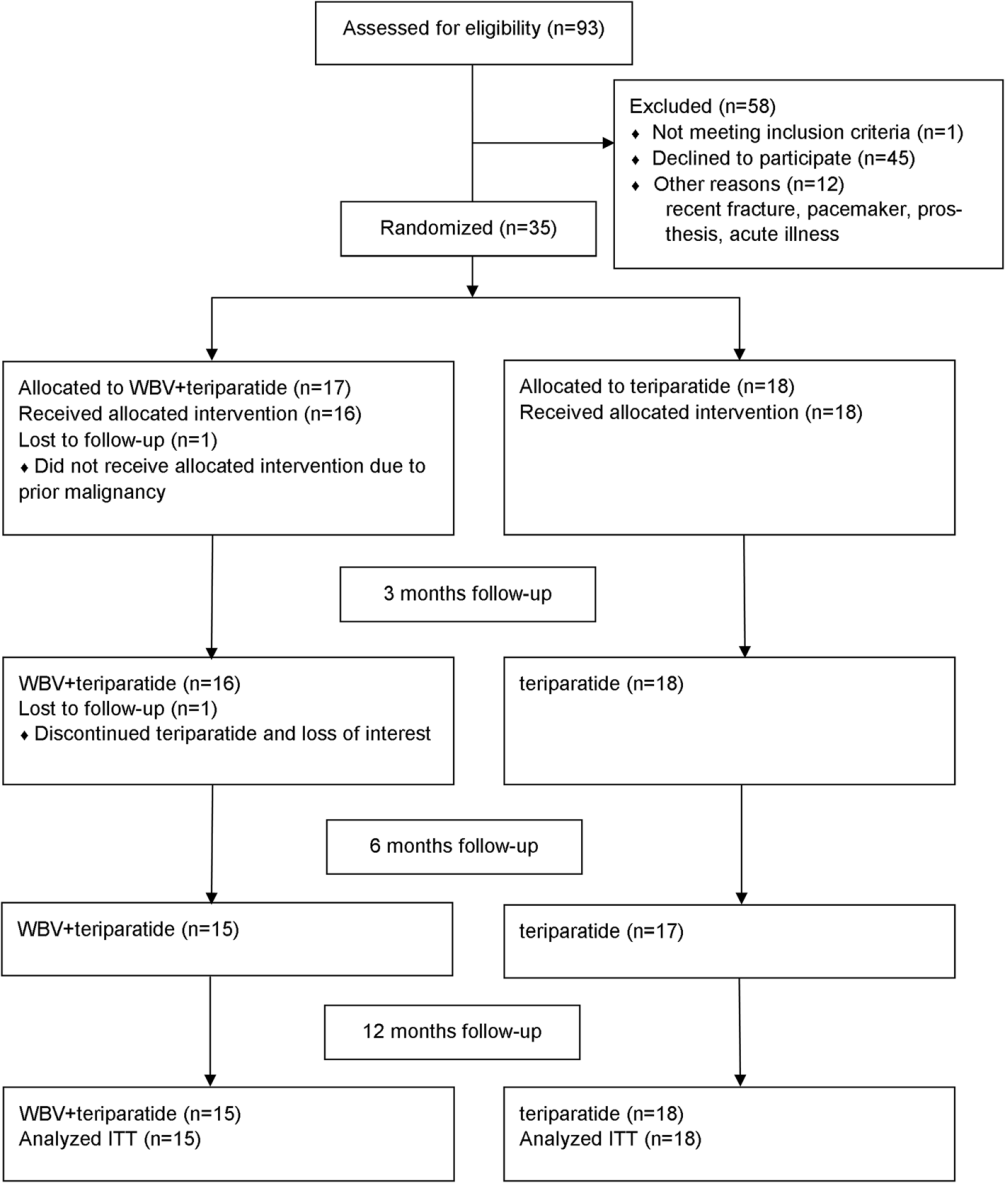
Consort flow diagram showing number of subjects screened for study participation and number of participants in each treatment group at baseline, three-, six-, and twelve months of follow-up, with designation of reasons for early discontinuation. The WBV + teriparatide group received whole-body vibration and teriparatide and the teriparatide group received teriparatide alone. WBV whole-body vibration, ITT intention to treat

The recruitment centers informed 93 eligible women who were starting teriparatide treatment about the study. A total of 35 postmenopausal women aged (mean ± SD) 69 ± 7 (range 53–81) years were included (Fig. 1). Of the 35 included participants 33 (94%) completed 3 months-, 32 (91%) completed 6 months-, and 32 (91%) completed 12 months examinations. In the WBV + teriparatide group, one participant never started teriparatide and was lost to follow-up after baseline measurements, one participant was lost to follow-up after 3 months, one participant did not attend 3 months follow-up but attended six- and 12 months follow-up, and two participants discontinued the WBV after 3 months but continued teriparatide and attended the follow-up visits. In the teriparatide group, one participant was lost to follow-up after 3 months. The consort flow diagram illustrates recruitment and participants’ flow through the study (Fig. 1). Baseline characteristics of the study population are shown in Table 1. There were no significant differences between the two groups.

**Table 1:** Baseline characteristics of study participants treated with whole-body vibration and teriparatide compared to teriparatide alone

	WBV + teriparatide group (*n* = 17)	teriparatide group (*n* = 18)	*P* value
Age (years) (mean ± SD)	69 ± 5	69 ± 8	0.909
BMI (kg/m^2^)(mean ± SD)	24.5 ± 4.1	24.1 ± 4.3	0.797
Charlson comorbidity score, median (IQR)			
Smoking, n (%)	1 (0–1)	0 (0–0)	0.087
Current	4 (24)	4 (22)	0.630
Former	6 (35)	9 (50)	
Never	7 (41)	5 (28)	
Physical activity at baseline*, n (%)			
Low	10 (59)	8 (39)	0.421
moderate	4 (23)	8 (50)	
High	3 (18)	2 (11)	
Falls in the last 12 months¤, n (%)			
None	10 (59)	11 (61)	0.244
1	3 (18)	6 (33)	
≥ 2	4 (24)	1 (6)	
Alcohol, (units a day)¤, n (%)			
none	3 (18)	6 (33)	0.543
up to 1	11 (64)	10 (56)	
> 1	3 (18)	2 (11)	
Ability to rise from chair without using arms ¤, n (%)	12 (71)	16 (89)	0.177

*P* values for difference t-test when normal distribution, chi squared for categorical, mann-whitney test for continual non-normal distribution.* Estimated using the IPAQ questionnaire, self-reported outcome. ¤ Self-reported outcome BMI body mass index, WBV whole-body vibration

### Physical performance and muscle function

The observed physical performance measurements are presented in Table 2.

**Table 2:** Physical performance measures at baseline, three, six, and twelve months of follow-up in patients treated with whole-body vibration and teriparatide (intervention) compared to teriparatide alone (control)

	Intervention	P change within group from baseline	Control	P change within group from baseline	P change between groups from baseline
SPPB, point	Mean ± sd		Mean ± sd		
Baseline	9.13 ± 1.96 *n* = 16		10.16 ± 1.82 *n* = 18		
Three months	10.35 ± 1.69 *n* = 14	0. 014*	10.17 ± 2.00 *n* = 18	1.000	0.065
Six months	9.46 ± 2.29 *n* = 15	0.128	10.17 ± 1.70 *n* = 17	0.742	0.372
Twelve months	9.57 ± 2.50 *n* = 14	0.043*	10.24 ± 1.82 *n* = 17	0.606	0.248
5xChairstand time, with arms, s					
Baseline	18.9 ± 7.9 *n* = 16		13.8 ± 4.6 *n* = 18		
Three months	14.4 **±** 6.9 *n* = 14	0.036*	13.0 ± 3.5 *n* = 18	0.395	0.117
Six months	16.2 ± 6.3 *n* = 15	0.085	13.4 ± 3.7 *n* = 17	0.572	0.185
Twelve months	15.7 ± 9.8 *n* = 14	0.040*	15.3 ± 6.6 *n* = 17	0.341	0.037
Time 3 m walk, s					
Baseline	2.9 ± 0.7 *n* = 17		2.8 ± 0.9 *n* = 18		
Three months	2.7 ± 0.5 *n* = 15	0.722	2.8 ± 0.8 *n* = 18	0.848	0.896
Six months	3.0 ± 1.0 *n* = 15	0.977	2.8 ± 0.9 *n* = 17	0.912	0.956
Twelve months	3.4 ± 1.8 *n* = 15	0.049*	2.7 ± 0.8 *n* = 17	0.622	0.077
Tandem total, s					
Baseline	27.8 ± 4.6 *n* = 17		28.8 ± 2.8 *n* = 18		
Three months	28.3 ± 2.5 *n* = 15	0.525	28.4 ± 3.0 *n* = 18	0.680	0.470
Six months	28.8 ± 2.4 *n* = 15	0.400	27.7 ± 3.1 *n* = 17	0.255	0.177
Twelve months	28.9 ± 2.3 *n* = 15	0.191	28.3 ± 2.9 *n* = 17	0.226	0.094
TUG, s					
Baseline	10.7 ± 3.2 *n* = 17		9.6 ± 3.1 *n* = 18		
Three months	9.2 ± 2.5 *n* = 15	0.257	9.2 ± 2.8 *n* = 18	0.580	0.650
Six months	10.0 ± 4.3 *n* = 15	0.395	9.3 ± 4.3 *n* = 17	0.668	0.746
Twelve months	11.4 ± 7.2 *n* = 15	0.394	8.95 ± 2.9 *n* = 17	0.344	0.205
Leg extension power, W/kg					
Baseline	0.91 ± 0.37 *n* = 17		1.10 ± 0.43 *n* = 18		
Three months	1.07 ± 0.42 *n* = 15	0.175	1.24 ± 0.48 *n* = 18	0.026*	0.606
Six months	1.13 ± 0.50 *n* = 15	0.010*	1.27 ± 0.64 *n* = 17	0.017*	0.798
Twelve months	1.08 ± 0.49 *n* = 15	0.044*	1.36 ± 0.53 *n* = 17	0.000*	0.339
Total Lean mass, kg					
Baseline	38.53 ± 6.56 *n* = 17		37.04 ± 3.88 *n* = 18		
Twelve months	38.20 ± 6.65 *n* = 15	0.281	37.04 ± 4.84 *n* = 17	0.657	0.276
Appendicular lean mass index kg/m^2^					
Baseline	5.89 ± 0.76 *n* = 17		6.10 ± 0.89 *n* = 18		
Twelve months	5.84 ± 0.66 *n* = 15	0.421	6.13 ± 0.92 *n* = 17	0.692	0.391

*P* values are derived from a mixed linear regression model with time, time x treatment interaction and adjustment for baseline group values, or robust cluster regression model with time, time x treatment and adjustment for baseline group values with clusters for repeated measurements.*Indicates a significant change *p* < 0,05. n = number participants, SPPB short physical performance battery, TUG timed up and go

The WBV + teriparatide group improved significantly in SPPB (mean ± SD) at 3 months compared to baseline from 9.13 ± 1.96 to 10.35 ± 1.69 (*p* = 0.014) (mainly driven by an improvement in 5TSTS) (Table 2), whereas no significant change was seen in the teriparatide group (10.16 ± 1.82 vs 10.17 ± 2.00). A trend towards a difference between-group in favor of the WBV + teriparatide group was observed for SPPB score at 3 months 0.86 [95%CI (− 0.05,1.77), *p* = 0.065]. Also, a post hoc analysis (including participants using their arms) showed a statistical significant between-group change in favor of the WBV + teriparatide group for 5TSTS score at 12 months − 4.7 s [95%CI (− 9.1,-0.3), *p* = 0.037]. The WBV + teriparatide group had a significantly higher 5TSTS time at baseline compared to the teriparatide group which was adjusted for in the linear regression model. The WBV + teriparatide group improved significantly in 5TSTS (mean ± SD) at three and 12 months of follow-up (18.9 ± 7.9 s, 14.4 ± 6.9 s, and 15.7 ± 9.8 s at baseline, 3 months, and 12 months, respectively (*p* < 0.05)). A sensitivity analysis excluding participants who used their arms for support revealed a between-group change score of − 7.3 s [95%CI (− 11.7, − 2.8), *p* = 0.002] at 12 months with a nonsignificant improvement in the WBV + teriparatide group at three- and 6 months and a significant improvement at 12 months (compared to no significant improvements in the teriparatide group). There were no significant difference between the groups in the time for walking three meters (mean ± SD); however, it increased significantly in the WBV + teriparatide group at 12 months (2.9 ± 0.7 at baseline vs 3.4 ± 1.8 at 12 months, *p* = 0.049) (Table 2). Both groups improved significantly in leg extension power during the study with no significant difference between groups (Table 2). No significant change in hand grip strength, TUG, total lean mass, or appendicular lean mass was observed at any time point in either group (Table 2). No statistical between-group differences were observed.

When missing data were replaced by multiple imputations the results were similar to those observed with the within-group improvement in SPPB in the WBV + teriparatide group at 3 months being + 0.89 [95%CI (0.06,-1.71), *p* = 0.035]; however, the between-group change in SPPB at 3 months were 0.89 [95%CI (− 0.19,1.97), *p* = 0.107].

Overall, the PP analysis demonstrated similar results as the ITT, and no changes between groups were statistically significant (data not shown). However, the PP analysis did show a significant improvement in TUG (mean ± SD) in the WBV + teriparatide group from 10.7 ± 3.7 s at baseline to 9.2 ± 2.8 s at 3 months (*p* = 0.045 for change within-group with no statistical significant difference between groups). There were no significant between group changes in self-reported physical activity (mean MET/min) from baseline to 12 months follow-up. However, both groups increased their activity levels (*p* < 0.05) (WBV + teriaparatide group: low = 59%, moderate = 23%, and high = 18% changed to low = 33%, moderate = 27%, and high = 40%) (Teriaparatide group: low = 39%, moderate = 40%, and high = 11% changed to low = 13%, moderate = 63%, and high = 25%).

### Adherence and adverse events

A total of 13 (76.5%) participants reported > 75% WBV training adherence (i.e. more than 685 sessions out of 913). Two participants reported pain in the lower extremities and one of them stopped the WBV prematurely without any reasons related to the WBV.

A total of 33 participants (94%) had adherence > 80% with the teriparatide treatment based on the percentage of collected prescribed prescriptions with no significant differences between groups. Also, no significant change in reported pain or dizziness was seen within- or between-groups. A few serious adverse events were reported: five hospital admissions (four in the teriparatide group and one in the WBV + teriparatide group), one distal femur fracture (WBV + teriparatide group), and one vertebral fracture (WBV + teriparatide group). No serious adverse events were believed to be related to WBV. In the 12 months of follow-up, two participants in the teriparatide group reported one fall each. In the WBV + teriparatide group, three participants fell once, one participant fell twice, and one participant fell three times. No falls occurred in relation to the training. There were no significant differences in fallers, falls, or adverse events between the two groups.

## Discussion

In this study that reports pre-specified secondary outcomes from a 12 months RCT comparing WBV and teriparatide with teriparatide alone in osteoporotic women some beneficial effects of WBV + teriparatide on physical performance were seen. The minimal clinically important change estimates for SPPB in older adults is 0.3–0.8 points and improvements of 0.4–1.5 points are associated with a substantial change in self-reported mobility [25]. The WBV + teriparatide group in our study had clinical relevant improvements in SPPB (0.86), mainly driven by improvements in the 5TSTS. Leg extension power improved statistically in both groups whereas no clinical or statistical improvements were observed in TUG, grip strength, or muscle mass.

While no prior studies have addressed the combined effect of WBV and teriparatide others have reported mixed results regarding the effects of WBV on physical parameters in postmenopausal women. One study found a positive effect of WBV on jumping height in postmenopausal women with osteoporosis compared to combined balance and resistance training [12], while a recent study did not find an effect of WBV on leg extension power or jumping performance in osteopenic postmenopausal women [26]. Another study using the same WBV training protocol as used in our study did however find a positive effect of WBV on leg extension power in older adults [10]. The specific WBV training protocol may be important because differences in duration, repetitions, vibration frequencies, and amplitude result in different training acceleration and training dosage. The improvements in SPPB in the WBV + teriparatide group in our trial were at its highest at 3 months of treatment but this effect was not fully sustained throughout the study. This might be due to the progression period of the training program in the first 2 months where the number of training sessions increased or due to a lower persistence with time. There was a trend towards a treatment and time interaction at 3 months; however, the trial had insufficient power to detect significant effects between groups. Indeed, a posteriori power and sample size evaluation for this follow-up study on secondary outcomes showed that a total of 64 participants (32 in each group) would have been needed to complete the trial in order to yield sufficient power (> 80%) to detect a between-group difference (*p* < 0.05) of the observed 0.86 SPPB points in the multilevel linear mixed regression analysis. The PP analysis showed similar results as the ITT but with a smaller sample size and no changes between groups were significant. The PP revealed a significant improvement in TUG in the WBV + teriparatide group at 3 months of follow-up although this effect was not sustained.

In our study both groups improved in the observed leg extension power, which could be due to clinical reasoning together with a potential learning effect of the test procedure or that the participants in the teriparatide group participated in physical activity not related to the study. We assessed the participants’ physical activity levels at baseline and 12 months of follow-up via the IPAQ questionnaire and found a significant increase in physical activity levels in both groups at 12 months with no significant difference in the changes between groups. Physical activity not related to the study could affect our results but it would be unethical to limit habitual physical activity in the teriparatide group in light of the recommendations of the importance of exercise in this population [27].

Our study was not powered nor aimed at investigating a combined effect of WBV and teriparatide on falls. However, the participants in the WBV + teriparatide group experienced more falls during the study period compared to the teriparatide alone group, and this might be explained partly by a higher comorbidity index in the former group (*p* = 0.087). Furthermore, the WBV + teriparatide group performed significantly worse in the 5TSTS at baseline (18.9 s versus 13.8 s in the WBV + teriparatide and teriparatide alone group, respectively). A 5TSTS test above 15 s has previously been shown to be a predictor of recurrent falls [28, 29]. This may have affected the results due to the former group being at higher risk of falls at baseline compared to the latter group. To study the effect on falls per se it would require a much larger trial. Nevertheless a recent meta-analysis showed that WBV can reduce fall rates in line with other fall preventive training programs [30, 31].

### Study limitations

Some limitations of our study design and protocol should be noted. The participants were not blinded to the WBV intervention; however, the assessors obtaining the physical measurements were blinded to group allocation. Furthermore, the training was conducted unsupervised at the participants’ own homes and we do not know if the participants followed the instructions even though monthly telephone contact was made. The “at home” training design was chosen to ensure high adherence and availability since known barriers to exercise are transportation and lack of time [32]. Adherence was assessed through training logbooks and this method has previously been reported to be reliable with an excellent overall intra-class correlation of 0.96 compared to electronic monitoring [33]. Finally, the sample size had low power to detect changes in the secondary outcomes of physical performance measures. Although we were able to detect a clinically relevant improvement in SPPB, no significant difference between groups were detected and our results should therefore be interpreted with caution.

The study was conducted according to guidelines, registered at the online registry clinicaltrials.gov, and the protocol was published with high internal validity in the study conduct. Of the informed eligible patients starting teriparatide approximately one out of three were included in the study. The included participants might differ from the overall patient population treated with teriparatide; however, age and comorbidity are comparable to patients from a large register study assessing all patients treated with teriparatide in during 2003–2010 [34] giving our study high external validity. However, we only included women in our study and the results may therefore not be generalizable to men. The results of this study highlight the feasibility of WBV training in osteoporotic women with a high attendance rate of 91% completing 12 months of follow-up. Future trials could investigate the effect of WBV in a fall preventive setting, combining treatment of osteoporosis and WBV to improve physical performance in a dual approach to lower the risk of fragility fracture.

## Conclusion

In conclusion, the results of the current study show that combining WBV with teriparatide in post-menopausal women with severe osteoporosis is feasible and capable of improving some aspects of physical performance. The clinically relevant improvements in SPPB were seen after 3 months of training but were not fully sustained for the remainder of the trial. Whether increasing WBV intensity and progression would lead to improved mobility and reduced falls risk needs further investigation.

## Data Availability

Data are available from the authors upon reasonable request and with permission of the Committees on Health Research Ethics, and the Data Protection Agency (2008-58-0035, 13/40496).

## Abbreviations

5TSTS: Five times sit to stand
BMD: Bone mineral density
DXA: Dual-energy X-ray absorptiometry
IPAQ: International physical activity questionnaire
ITT: Intention to treat
PP: Per protocol
RCT: Randomized controlled trial
SPPB: Short physical performance battery
TUG: Timed-up-and-go
WBV: Whole-body vibration exercise
